# Effects of acute transcutaneous vagus nerve stimulation on emotion recognition in adolescent depression

**DOI:** 10.1017/S0033291719003490

**Published:** 2021-02

**Authors:** Julian Koenig, Peter Parzer, Niklas Haigis, Jasmin Liebemann, Tamara Jung, Franz Resch, Michael Kaess

**Affiliations:** 1 Section for Experimental Child and Adolescent Psychiatry, Department of Child and Adolescent Psychiatry, Centre for Psychosocial Medicine, University of Heidelberg, Blumenstr. 8, 69115 Heidelberg, Germany; 2 University Hospital of Child and Adolescent Psychiatry and Psychotherapy, University of Bern, Stöckli, Bolligenstrasse 141c, 3000 Bern 60, Switzerland; 3 Clinic for Child and Adolescent Psychiatry, Centre for Psychosocial Medicine, University of Heidelberg, Blumenstr. 8, 69115 Heidelberg, Germany; 4 Section for Translational Psychobiology in Child and Adolescent Psychiatry, Department of Child and Adolescent Psychiatry, Centre for Psychosocial Medicine, University of Heidelberg, Blumenstr. 8, 69115 Heidelberg, Germany

**Keywords:** Adolescents, depression, emotion recognition, vagus nerve stimulation

## Abstract

**Background:**

Transcutaneous vagus nerve stimulation (tVNS) is a promising therapeutic option for major depressive disorder (MDD) in adults. Alternative third-line treatments for MDD in adolescents are scarce. Here we aimed to assess the effects of acute tVNS on emotion recognition in adolescents with MDD.

**Methods:**

Adolescents (14–17 years) with MDD (*n* = 33) and non-depressed controls (*n* = 30) received tVNS or sham-stimulation in a cross-sectional, case–control, within-subject cross-randomized controlled trial, while performing different tasks assessing emotion recognition. Correct responses, response times, and errors of omission and commission on three different computerized emotion recognition tasks were assessed as main outcomes. Simultaneous recordings of electrocardiography and electro dermal activity, as well as sampling of saliva for the determination of *α*-amylase, were used to quantify the effects on autonomic nervous system function.

**Results:**

tVNS had no effect on the recognition of gradually or static expressed emotions but altered response inhibition on the emotional Go/NoGo-task. Specifically, tVNS increased the likelihood of omitting a response toward *sad* target-stimuli in adolescents with MDD, while decreasing errors (independent of the target emotion) in controls. Effects of acute tVNS on autonomic nervous system function were found in non-depressed controls only.

**Conclusions:**

Acute tVNS alters the recognition of briefly presented facial expressions of *negative* valence in adolescents with MDD while generally increasing emotion recognition in controls. tVNS seems to specifically alter early visual processing of stimuli of negative emotional valence in MDD. These findings suggest a potential therapeutic benefit of tVNS in adolescent MDD that requires further evaluation within clinical trials.

## Introduction

Major depressive disorder (MDD) is one of the leading causes of disability worldwide (Vos et al., 2016). Adolescence is a critical period for the development of depression with a cumulative incidence for first-onset depression of about 36% among female and 14% among male teenagers (12–17 years of age; Breslau et al., 2017). The worldwide prevalence of any depressive disorder in this age group is estimated at 2.6% (Polanczyk, Salum, Sugaya, Caye, & Rohde, 2015). Around 40% of adolescents do not benefit from the available first- and second-line treatments (i.e. psychotherapy and/or pharmacotherapy; Birmaher & Brent, 2007). Vagus nerve stimulation (VNS) is a promising third-line therapeutic option for treatment-resistant depression in adults (Aaronson et al., 2017). Recent technological advances allow for the transcutaneous stimulation of the vagus nerve (tVNS), minimizing the risk associated with surgery for implanting the stimulator. In adults, tVNS has been shown to reduce depressive symptoms following 4 weeks of stimulation (Fang et al., 2016). The potential of tVNS in the treatment of underage patients with depression has not yet been explored.

In a pre-clinical experimental trial, we aimed to investigate the potential effects of acute tVNS on neuropsychological proxies of MDD, namely emotion recognition. Deficits in recognizing facial expressions of emotional valence across all basic emotions except sadness have been repeatedly reported in depressed patients (Dalili, Penton-Voak, Harmer, & Munafò, 2015). Regarding sad facial expressions, patients with MDD show a *negativity bias* (e.g. tendency to rate natural stimuli as negative). In adults, selective serotonin reuptake inhibitors (SSRI) have been shown to improve emotion recognition (Harmer et al., 2003) and changes in processing facial expressions following SSRI administration have been shown to predict later treatment outcome (Godlewska, Browning, Norbury, Cowen, & Harmer, 2016). Behavioral data in healthy adults suggest similar effects of tVNS action as tVNS enhances the recognition of emotions in faces (Sellaro, de Gelder, Finisguerra, & Colzato, 2018) and promotes the ability to decode salient social cues (Colzato, Sellaro, & Beste, 2017). In a very first study with underage depressed patients, we aimed to study the potential antidepressant effects of tVNS by assessing its acute effects on emotion recognition.

## Materials and methods

### General procedures

**Fig. 1.**
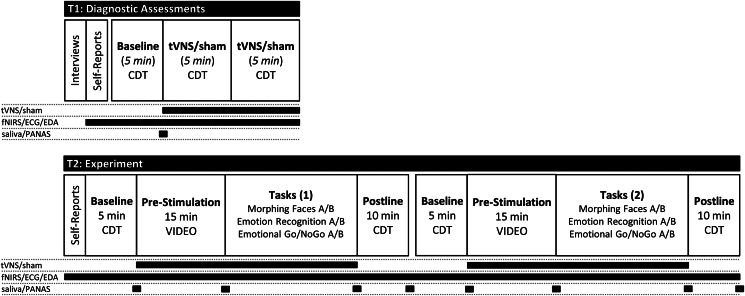
Illustration of the study design; the study comprised two appointments. Diagnostic assessments were conducted at T1. The actual experiment, including the neuropsychological tasks, was conducted at T2. Illustrated are the tVNS/sham stimulation periods (randomized order across participants); recording periods for electrocardiography (ECG), electrodermal activity (EDA) as well as function near infrared spectroscopy (fNIRS); and time points of saliva sampling and self-reports on current affective states (PANAS).

The ethics committee of the Medical Faculty, Heidelberg University approved the study (Study ID: S-297/2016 and S-365/2017). The trial was registered at the *German Clinical Trials Register* (Study ID: DRKS00011112) and the *World Health Organization* (Universal Trial Number: U1111-1188-0829). Adolescent patients with MDD during a current depressive episode were recruited consecutively at the Clinic for Child and Adolescents Psychiatry, Heidelberg University between December 2016 and October 2017. Non-depressed controls were recruited between October 2017 and January 2018 via public advertisement. Due to ethical and legal restrictions, non-depressed controls were adolescents fulfilling diagnostic criteria for tension-type headache (TTH). The tVNS device used (*see below*) has a CE marking for the treatment of depression and pain in accordance with EU regulations regarding conformity with health, safety, and environmental protection standards. Thus, we were not allowed to conduct an experimental trial with completely *healthy* controls, not fulfilling the area of intended indication according to CE criteria. All participants and their legal guardians provided written informed consent before inclusion in the study. Participants received an allowance of €50 for participation. Details on the inclusion and exclusion criteria as well as the participant flow are reported in the online Supplementary Material. The general study design is illustrated in Fig. 1.

### Clinical assessments and self-reports

Following an initial screening, participants were informed about the study details. In case written informed consent was obtained, participants were assigned a study ID and invited to a first appointment including all clinical assessments (T1). At T1, participants provided basic sociodemographic information before completing several clinical interviews. All assessments were parallelized across groups. Clinical interviews included the German version of the *Mini-International Neuropsychiatric Interview for Children and Adolescents* (M.I.N.I – KID 6.0; Sheehan et al., 2010). Patients had to endorse diagnostic criteria for MDD with a current depressive episode. Non-depressed controls were only included if they did not endorse any primary psychiatric disorder requiring treatment or any current psychiatric or psychological treatment. Participants completed the *Children*'*s Depression Rating Scale – Revised* (CDRS-R; Poznanski, Freman, & Mokros, 1985). Only patients scoring above the severity cut-off score of 34 were included in the trial. Controls scoring above the cut-off were excluded. Clinicians rated the level of emotional and behavioral functioning in the past 3 months using the German version of the Children's Global Assessment Scale (C-GAS; Shaffer et al., 1983). Participants were asked to complete several self-reports, including the Difficulties in Emotion Regulation Scale (DERS; Gratz & Roemer, 2004), and the Beck Depression Inventory II (BDI-II; Beck, Steer, & Brown, 1996; Osman, Kopper, Barrios, Gutierrez, & Bagge, 2004). At T2, patients further completed the State Trait Anxiety Inventory (STAI; Laux, Glanzmann, Schaffner, & Spielberger, 1981). The assessment further included other clinical interviews and self-reports (details reported in the online Supplementary Material), not reported in the present manuscript. All interviews and self-reports were computerized using *LimeSurvey*.

### Neurobiological measures

Following interviews and self-reports at T1, functional near-infrared spectroscopy (fNIRS^†^^1^) of the prefrontal cortex, electrocardiography (ECG), and electrodermal activity (EDA) were recorded during a 5 min baseline while participants completed a Color Detection Task (CDT; Jennings, Kamarck, Stewart, Eddy, & Johnson, 1992; see online Supplementary Material). After the baseline, a saliva sample was taken. Time stamps for all events were recorded on computerized paper (TeleForm–Electric Paper). Participants then received 5 min of tVNS and sham stimulation in randomized order while fNIRS, ECG, and EDA were continuously recorded. At the end of T1, weight and height were measured. Data from physiological recordings at T1 are not reported. Following T1, eligible patients were invited to a second appointment. All appointments for T1 and T2 were scheduled in the afternoon (past 1 pm). After completion of self-reports, equipment for the recording of fNIRS, ECG, and EDA was attached. Patients again first completed a 5 min baseline (CDT) after which the tVNS stimulator was attached. Patients then completed a 15 min pre-stimulation phase (tVNS or sham) while watching a video (see online Supplementary Material) followed by the first task period, and a 10 min postline (CDT). Baseline (5 min), pre-stimulation (tVNS or sham; 15 min video), task-period, and post-line (10 min, CDT) were repeated before participants were debriefed. fNIRS, ECG, and EDA were continuously recording throughout all procedures at T2. Participants provided saliva samples after each baseline, after each pre-stimulation period, after each task-period, and after each postline. While providing saliva samples, participants completed self-reports on positive and negative affect using a computerized short version of the Positive and Negative Affect Schedule (PANAS; Watson, Clark, & Tellegen, 1988) and rated their perceived stress and current mood on 100 mm visual analogue scales (VAS). Focusing on measures quantifying physiological effects of tVNS on autonomic nervous system activity, saliva samples were used for the assay of *α*-amylase. ECG data were analyzed to quantify heart rate variability (HRV) and mean heart rate (HR) for each segment. EDA data were analyzed to derive skin conductance response (SCR). Here we only report on *α*-amylase and SCR as proxies of sympathetic activity, HRV as a proxy of parasympathetic activity and HR (mixed influence). fNIRS was recorded as an additional measure to quantify oxygenation in the prefrontal cortex (not reported). Details on the recording and analyses of all neurobiological measures are provided in the online Supplementary Material.

### Transcutaneous vagus nerve stimulation

**Fig. 2.**
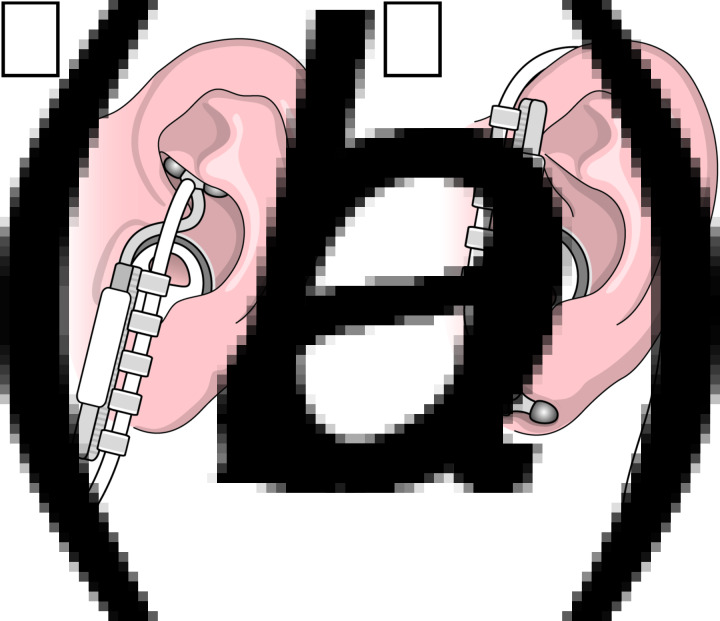
Schematic illustration of the placement of the ear electrode; (*a*) active transcutaneous vagus nerve stimulation; the ear electrode is placed in contact with the skin of the left concha; (*b*) sham stimulation; the ear electrode is placed in contact with the left ear lobe, not innervated by the vagus nerve.

tVNS was applied using the VITOS^®^ t-VNS device (Cerbomed, Erlangen, Germany). The VITOS^®^ is a battery powered handheld stimulator with an ear electrode stimulating at the concha of the outer ear. It has a CE mark for the treatment of pain and depression (CE0408). Stimulation is achieved via a series of electrical pulses with a pulse width of 250 µs at a frequency of 1 Hz with a cycle of 30 s on and 30 s off to avoid habituation. The stimulation intensity for all participants and both conditions (tVNS and sham) was set to 0.5 mA. Before placement of the ear electrode, the left concha and ear lobe were cleaned using alcohol swipes (70% isopropyl alcohol). During active tVNS, the ear electrode was placed in contact with the skin of the left concha, as illustrated in Fig. 2*a*. For sham stimulation, the ear electrode was placed in contact with the left ear lobe, not innervated by the vagus nerve (Burger et al., 2016; Colzato et al., 2017; Frangos, Ellrich, & Komisaruk, 2015) as illustrated in Fig. 2*b*. The device automatically measures impedance and insufficient contact of the electrode with the skin evokes an alarm. tVNS or sham stimulation were applied in a within-subject cross-randomized order. tVNS or sham stimulation started 15 min (pre-stimulation) before participants were asked to complete the neuropsychological tasks.

### Neuropsychological tasks

A total of three computerized neuropsychological tasks were used to quantify different facets of emotion recognition. All tasks were programmed using PsychoPy (version: 1.84; Peirce, 2007). Stimuli for the different tasks were taken from the FACES database, a database of facial expressions in younger, middle-aged, and older women and men (Ebner, Riediger, & Lindenberger, 2010). Only faces of younger and middle-aged women and men were used (the tasks including lists of stimuli are available online: https://osf.io/pjnhx/). All tasks were programmed in two versions (A/B) for repeated presentation, using distinct sets of stimuli. The order of tasks was kept constant, while the order of versions was randomized. Stimuli were randomly assigned to the different tasks to avoid the repeated presentation of the same faces, except for the emotional Go/NoGo-task. Age (younger and middle-aged) and sex (women and men) of faces were equally balanced across tasks, versions (A/B), and emotional expressions. The reporting of all methodological details of the tasks is provided in the online Supplementary Material. First, patients were asked to complete a task assessing the capacity to recognize emotions (happy, sad, anger, fear) in gradually changing facial expressions (starting from neutral toward the target emotion). Patients were instructed to press the mouse key once they recognize the expressed emotion. The response time (time until recognition of emotional expression and selection of one category) in ms, the level of emotion expression at response in percent, and the selected category (correct responses) were recorded. Second, patients were asked to complete a basic emotion recognition task. Participants were asked to select the category of the expressed static emotion (happy, neutral, sad), and in case participants selected one of the discrete emotional categories (happy or sad), they were asked to rate the intensity of the expressed emotion (‘How intense is the expressed emotion?’) on a 100 mm VAS. The response time (time until selection of one category) in ms, the selected category (correct responses), and the rating of emotional intensity (0–100; in case *happy* or *sad* were selected as category) were recorded. Third, patients were asked to complete an emotional Go/NoGo-task designed in accordance with a previous study (Trinkl et al., 2015). Following an instruction screen, patients were asked to press the left mouse key whenever a target stimulus was displayed (go-cue) and inhibit the response whenever a non-target stimulus was displayed (no-go-cue). Each version of the task (A/B) consisted of two blocks. Across blocks go- and no-go-cues were interchanged. The number of correct and false hits, as well as the reaction time in ms, was recorded.

### Statistical analyses

Differences between groups on sociodemographic and clinical variables were analyzed using χ^2^ (categorical variables) or *t* tests (continuous variables), respectively. Neuropsychological task data were analyzed using multilevel mixed-effects generalized linear models for binominal data (dichotomous outcomes) or multilevel mixed-effects linear regression (continuous outcomes). Group (MDD *v.* controls) and condition (tVNS *v.* sham) as well as their interaction were addressed as fixed effects. The participants' ID was entered as a random effect. In the case of a significant group by condition interaction, we further tested for the effect of target emotion (e.g. sad *v.* happy) in each level of the interaction. Self-reports (stress, mood, positive, and negative effect) as well as physiological data (HR, HRV, SCR, *α*-amylase) were analyzed using multilevel mixed-effects linear regression with time (segment or time of measurement), group, and condition as fixed effects and the participants' ID as random effect. Based on reviewer recommendations, we performed additional analyses. (1) In line with previous tVNS research (Colzato et al., 2017; Sellaro et al., 2018), we conducted additional analyses addressing a potential interaction of tVNS stimulation with item difficulty (i.e. recognizability of faces). Data on item difficulty were derived from a validation study as provided on the FACES website (http://faces.mpdl.mpg.de/imeji/). In line with previous studies (Colzato et al., 2017; Sellaro et al., 2018), item difficulty was dichotomized to compare *easy* and *difficult* items. (2) In exploratory analyses, we addressed the linear association between effects seen under tVNS and measures of clinical severity on a continuum using Pearson's correlations (two sided). All analyses were performed using Stata (Version 15.1; StataCorp LP, College Station, TX, USA), at an *α* level of 0.05. All contrasts were Sidak corrected.

## Results

### Sample characteristics

Groups did not differ on sex, age, height, weight, or school-type. Groups differed on all clinical variables, with MDD patients reporting greater depression severity, greater difficulties in emotion regulation, state and trait anxiety, as well as a lower level of functioning. Detailed reporting on group differences in sociodemographic and clinical variables is provided in Table 1.

**Table 1.** Sociodemographic and clinical characteristics by group

	MDD	Controls	*P*
*N* (female %)	33 (81.82)	30 (80.00)	0.854
Height, cm	167.42 (8.80)	167.83 (8.95)	0.856
Weight, kg	63.03 (13.73)	59.40 (1.42).207	0.207
School, *n* (%)			0.346
Hauptschule	2 (6.06)	0 (0.00)	
Realschule	11 (33.34)	8 (26.67)	
Gymnasium	15 (45.45)	19 (63.34)	
Other	5 (15.15)	3 (10.00)	
MDD DSM-5, *n* (%)			
Current	33 (100.00)	0 (0.00)	<0.0001
Past	29 (87.88)	3 (10.00)	<0.0001
Recurrent	9 (27.27)	0 (0.00)	<0.0001
Medication, *n* (%)			
Fluoxetine	4 (12.12)	0 (0.00)	
Mirtazapin	2 (6.06)	0 (0.00)	
Sertralin	1 (3.03)	0 (0.00)	
Other	2 (6.06)	1 (3.33)	
CDRS	73.39 (10.59)	19.93 (10.59)	<0.0001
BDI	36.00 (12.37)	4.67 (4.57)	<0.0001
DERS	117.45 (23.90)	61.57 (12.39)	<0.0001
STAI (state)	52.48 (8.16)	32.27 (6.03)	<0.0001
STAI (trait)	61.24 (7.81)	33.07 (8.24)	<0.0001
CGAS	44.61 (7.74)	93.23 (6.79)	<0.0001
Comorbidity (ICD-10), *n* (%)			
F0X	0 (0.00)	0 (0.00)	
F1X	12 (36.36)	4 (13.33)*	0.036
F2X	2 (6.06)	0 (0.00)	0.171
F3X	33 (100.00)	3 (9.09)	<0.0001
F4X	24 (72.72)	2 (6.06)	<0.0001
F5X	7 (21.21)	0 (0.00)	0.007
F6X (other than BPD)	0 (0.00)	0 (0.00)	
F7X	0 (0.00)	0 (0.00)	
F8X	0 (0.00)	0 (0.00)	
F9X	13 (39.39)	1 (3.03)	0.001

All values are means and standard deviations (s.d.) in brackets unless otherwise indicated; MDD, major depressive disorder; school, after 4 years of elementary school the German school system branches into three types of secondary schools. The so-called *Hauptschule* (Secondary General School which takes 5 years after Primary School) prepares pupils for vocational training, whereas the *Realschule* (Intermediate Secondary School) concludes with a general certificate of secondary education after 6 years. Eight years of Gymnasium provide pupils with a general university entrance qualification; medication: multiple counts possible, data on doses and duration of intake available upon request; CDRS, Children's Depression Rating Scale – Revised; BDI, Beck Depression Inventory II; DERS, Difficulties in Emotion Regulation Scale; STAI, State Trait Anxiety Inventory; CGAS, Children's Global Assessment Scale; no missing data; *comorbidity in controls: harmful use of alcohol (F10.1; *n* = 4); remitted depressive disorder (F33.4; *n* = 3); social phobia (F40.1; *n* = 2); agoraphobia with panic disorder (F40.01; *n* = 1).

### Self-reported mood and stress

Positive affect [χ^2^_(7)_ = 97.36, *p* *<* 0.0001], negative affect [χ^2^_(7)_ = 21.22, *p* = 0.004], stress [χ^2^_(7)_ = 44.52, *p* *<* 0.0001], and current mood [χ^2^_(7)_ = 27.66, *p* *<* 0.001] varied over time. Further, positive affect [χ^2^_(1)_ = 34.94, *p* *<* 0.0001], negative affect [χ^2^_(1)_ = 21.14, *p* *<* 0.0001], stress [χ^2^_(1)_ = 37.74, *p* *<* 0.0001], and current mood [χ^2^_(1)_ = 74.13, *p* *<* 0.0001] differed as a function of group, indicating less stress and negative affect as well as increased positive affect and current mood in controls compared to patients with MDD. There were no significant main effects of tVNS. There was a significant group by tVNS interaction on ratings of current mood [χ^2^_(1)_ = 9.27, *p* = 0.002], such that mood slightly decreased (−3.45 points on the VAS) under tVNS in patients with MDD, mainly in the respective baseline and pre-stimulation phases. There were no effects in controls. Findings are illustrated in online Supplementary Fig. S1.

### Emotion recognition

Groups significantly differed in the time needed to classify and correctly classify gradually expressed emotions (Task 1). Controls were faster in classifying gradually changing facial expressions of emotions. Similarly, groups significantly differed in the time needed to classify and correctly classify static expressed emotions (Task 2). Controls were faster in classifying static facial expressions of emotions. There were no effects of tVNS on the recognition of static or gradually expressed emotions (see Table 2).

**Table 2.** Descriptive statistics on neuropsychological task outcomes by group and condition

	tVNS		Sham		Group		tVNS		Interaction	
	MDD	Controls	MDD	Controls	χ^2^	*p*	χ^2^	*p*	χ^2^	*p*
Task 1: morphing faces										
Hits (%)	86.74 (12.74)	84.69 (15.05)	87.22 (12.36)	86.98 (13.69)	0.01	0.923	1.80	0.179	0.77	0.379
Response time (*sec*)	8.97 (2.70)	7.72 (2.34)	9.01 (2.70)	8.02 (2.21)	4.21	0.040	0.32	0.574	0.20	0.657
Response time (*sec*) correct trials	8.97 (2.47)	7.73 (2.27)	8.92 (2.69)	8.0 (2.05)	4.15	0.042	0.18	0.674	0.38	0.535
Task 2: emotion recognition										
Hits (%)	94.57 (6.29)	94.44 (8.25)	96.39 (3.41)	96.81 (4.98)	1.47	0.225	0.08	0.780	0.18	0.676
Response time (*sec*)	3.18 (0.78)	2.74 (0.60)	3.10 (0.67)	2.95 (0.66)	5.07	0.024	0.45	0.502	1.78	0.183
Response time (*sec*) correct trials	3.10 (0.75)	2.67 (0.50)	3.01 (0.60)	2.90 (0.61)	4.91	0.027	0.56	0.454	2.97	0.085
Intensity (*VAS*)	56.60 (12.50)	59.20 (12.99)	56.39 (11.44)	62.45 (12.90)	2.30	0.129	1.63	0.202	2.11	0.146
Intensity (*VAS*) correct trials	59.54 (13.65)	61.04 (13.76)	59.61 (12.81)	64.27 (13.21)	1.01	0.316	1.75	0.186	1.59	0.208
Task 3: emotional Go/NoGo										
Hits (%)	84.00 (13.18)	90.86 (5.83)	85.96 (10.56)	87.47 (13.07)	4.47	0.036	4.49	0.034	29.76	<0.0001
Omission errors (%)	7.60 (10.47)	2.73 (2.94)	5.52 (7.49)	5.73 (9.91)	4.24	0.040	8.36	0.004	59.25	<0.0001
Commission errors (%)	8.40 (4.77)	6.41 (4.39)	8.52 (4.68)	6.80 (4.39)	3.19	0.074	0.43	0.510	0.16	0.687
Response time (*sec*)	0.40 (0.07)	0.4 (0.07)	0.39 (0.08)	0.42 (0.06)	1.10	0.295	0.35	0.556	2.69	0.101
Response time (*sec*) correct trials	0.40 (0.07)	0.41 (0.07)	0.40 (0.08)	0.42 (0.06)	1.06	0.303	0.90	0.343	1.62	0.203

MDD, major depressive disorder; tVNS, transcutaneous vagus nerve stimulation; sham, sham stimulation; hits, correct responses/correctly classified emotions; response time, time until response/recognition; intensity, ratings of intensity for discrete emotions on a visual analogue scale (VAS) 0–100; correct trials, only considering data when the emotion was correctly classified; omission errors, no reaction toward a target stimulus (go-trial); commission error, reaction toward a non-target stimulus (nogo-trial); percentage measures on correct hits only provided for descriptive purposes, analyses were based on dichotomous data nested by subject and trial using multilevel mixed-effects generalized linear models.

**Fig. 3.**
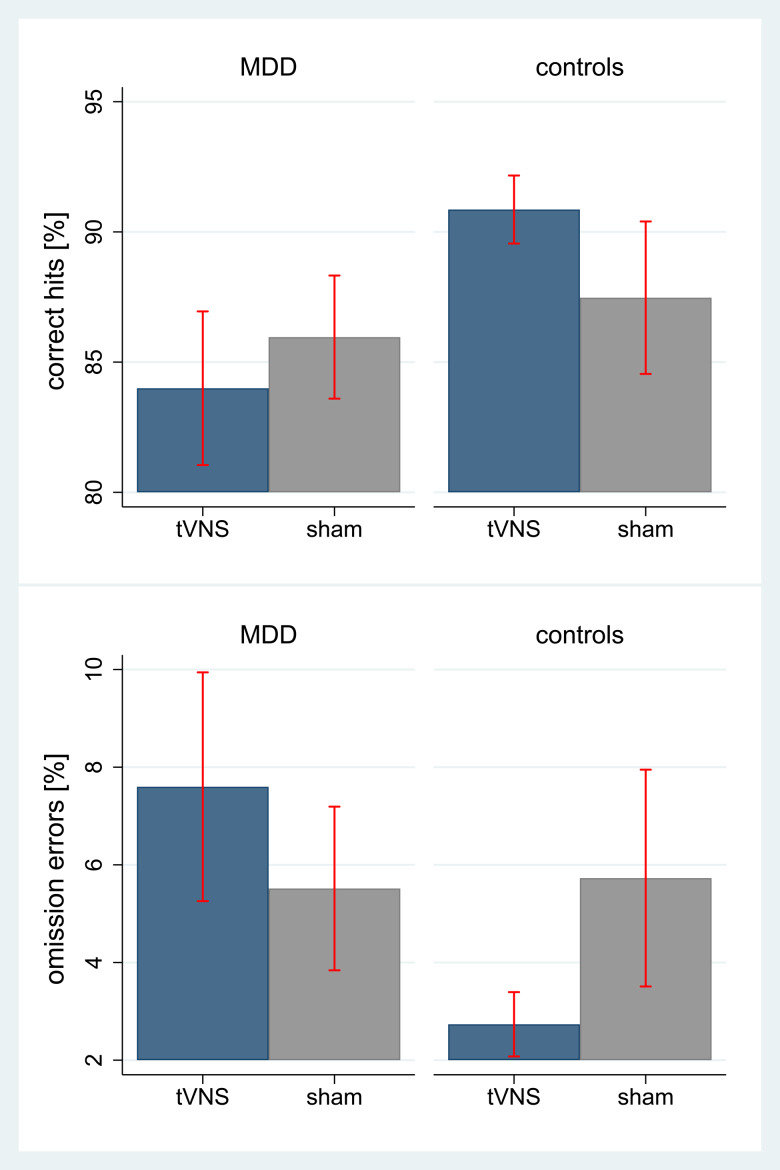
Main effects of tVNS on emotion recognition (hits and omission errors) in the emotional Go/NoGo-task by group; hits, correct responses toward target-stimuli in percent (total of 128 stimuli per condition); omission errors, no reaction toward a target stimulus (go-trial) in percent; tVNS, transcutaneous vagus nerve stimulation at the concha of the left outer ear; sham, sham-stimulation of the left ear lobe; MDD, adolescents with major depression; control, non-depressed adolescents; illustrated are means and 95% confidence intervals.

Groups significantly differed on correct responses and omission errors in the Go/NoGo-task. Controls were more likely to respond correctly and made less omission errors. tVNS had a significant effect on correct responses and omission errors, such that sham stimulation was associated with less correct responses and more omission errors. Further, there was a significant group by tVNS interaction on correct responses and omission errors, such that correct responses increased under tVNS in controls, while decreasing under tVNS in patients with MDD. Omission errors increased under tVNS in patients with MDD and decreased under tVNS in controls (Table 2), as illustrated in Fig. 3.

**Fig. 4.**
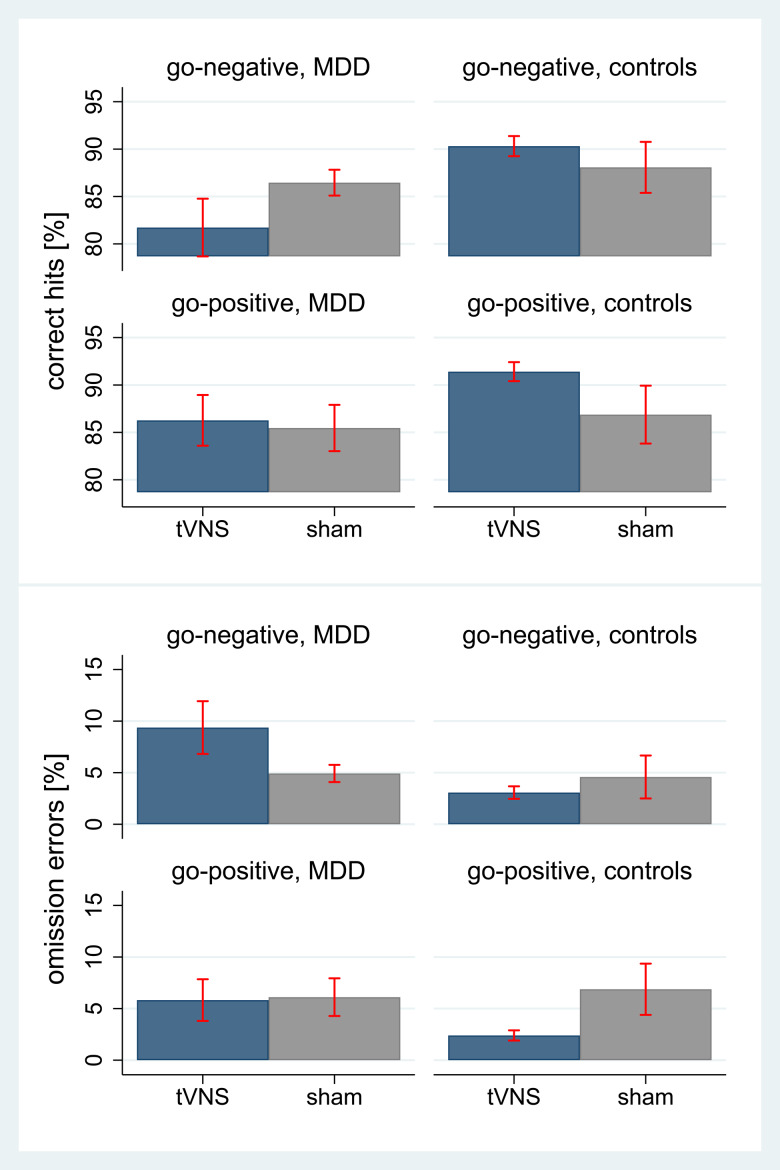
Effects of tVNS on emotion recognition (hits and omission errors) in the emotional Go/NoGo-task by target emotion and group; hits, correct responses toward target-stimuli in percent (total of 64 stimuli per condition and emotion); omission errors, no reaction toward a target stimulus (go-trial) in percent (%); tVNS, transcutaneous vagus nerve stimulation at the concha of the left outer ear; sham: sham-stimulation of the left ear lobe; MDD, adolescents with major depression; control, non-depressed adolescents; go-negative, target stimuli presenting faces with sad expressions; go-positive, target stimuli presenting faces with *happy* expressions; illustrated are means and 95% confidence intervals.

Analysis in each level of the significant group by tVNS interaction showed that the effects differed as a function of target emotion. In patients with MDD, correct hits on *sad* emotions decreased under tVNS (go-negative: *z* = −4.36, *p* *<* 0.0001) and no effects occurred for *happy* faces (go-positive: *z* = 0.78, *p* = 0.437). In controls, correct hits on *happy* (go-positive: *z* = 4.63, *p* *<* 0.0001) and *sad* emotions (go-negative: *z* = 2.31, *p* = 0.021) increased under tVNS. Omission errors on *sad* target stimuli (go-negative: *z* = 5.79, *p* *<* 0.0001) increased under tVNS in patients with MDD, and showed no differences for *happy* target stimuli (go-positive: *z* = −0.41, *p* = 0.685). In controls, omission errors decreased on *sad* (go-negative: *z* = −2.50, *p* = 0.012) and *happy* (go-positive: *z* = −6.56, *p* *<* 0.0001) target emotions under tVNS. Findings are illustrated in Fig. 4.

### Autonomic nervous system function

*α*-amylase [χ_(7)_ = 26.58, *p* *<* 0.001] changed over time but there were no significant main effects of group [χ_(1)_ = 1.66, *p* *<* 0.198] or condition [χ_(1)_ = 1.62, *p* *<* 0.202]. Both HR [χ_(17)_ = 376.03, *p* *<* 0.0001] and HRV [χ_(17)_ = 249.97, *p* *<* 0.0001] changed over time. HR [χ_(1)_ = 6.92, *p* = 0.009] and HRV [χ_(1)_ = 15.21, *p* *<* 0.0001] showed main effects of group, indicating lower HR and greater HRV in controls compared to patients with MDD. HR [χ_(1)_ = 0.72, *p* = 0.395] and HRV [χ_(1)_ = 2.03, *p* = 0.154] showed no main effects of tVNS. However, there were significant tVNS by group interactions on HR [χ_(1)_ = 13.33, *p* < 0.001] and HRV [χ_(1)_ = 14.33, *p* < 0.001]. HR decreased and HRV increased in controls under tVNS. There were no effects in patients with MDD. SCR changed over time [χ_(17)_ = 119.73, *p* < 0.0001], but showed no significant main effect of group [χ_(1)_ = 0.15, *p* = 0.694] or tVNS [χ_(1)_ = 0.35, *p* = 0.553]. Findings are illustrated in online Supplementary Fig. S2.

### Additional analyses: item difficulty and clinical severity^2^

Item difficulty (easy *v.* difficult) had a significant main effect on correct responses [χ_(1)_ = 4.86, *p* = 0.027] and time to correctly identify emotions [χ_(1)_ = 4.99, *p* = 0.025] in the recognition of gradually expressed emotions. Similarly, item difficulty had a significant main effect on correct responses when identifying static expressed emotions [χ_(1)_ = 5.00, *p* = 0.025] and on the perceived intensity of expressed emotions [all items: χ_(1)_ = 32.71, *p* *<* 0.0001; correctly identified items: χ_(1)_ = 50.57, *p* *<* 0.0001]. However, item difficulty showed no significant interaction with stimulation condition (tVNS *v.* sham) or group (MDD *v.* controls). Item difficulty had no significant effect on any outcome of interest in the emotional Go/NoGo task.

**Fig. 5.**
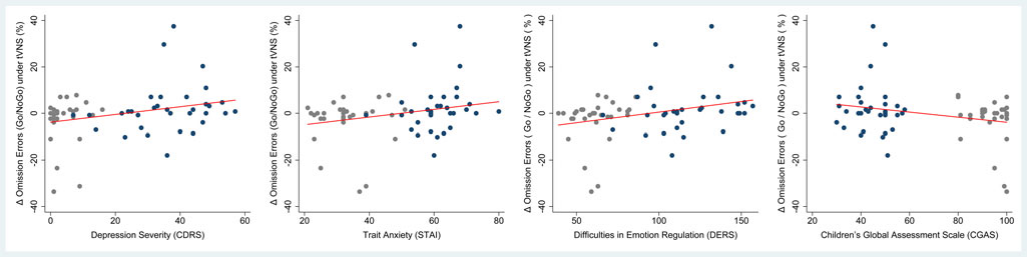
Linear associations between effects of tVNS and measures of a clinical outcome; illustrated are changes in omission errors under tVNS (delta score: sham – tVNS) in association with the respective clinical outcome in the full sample. omission errors, no reaction toward a target stimulus (go-trial) in percent (%); CDRS, Children's Depression Rating Scale – Revised; BDI, Beck Depression Inventory II; DERS, Difficulties in Emotion Regulation; CGAS, Children's Global Assessment Scale; blue dots: patients with MDD, gray dots: non-depressed controls.

Finally, we addressed the linear association between effects seen under tVNS in the emotional Go/NoGo task and measures of clinical severity. We found a significant association between the change in correct responses under tVNS and depression severity [CDRS interview: *r*_(63)_ = −0.272, *p* = 0.031; BDI self-reports: *r*_(63)_ = −0.253, *p* = 0.046], as well as self-reports on difficulties in emotion regulation [DERS: *r*_(63)_ = −0.261, *p* = 0.039]. Regarding omission errors, we found similar associations between changes under tVNS and depression severity [CDRS interview: *r*_(63)_ = 0.319, *p* = 0.011; BDI self-reports: *r*_(63)_ = 0.289, *p* = 0.021], difficulties in emotion regulation [DERS: *r*_(63)_ = 0.294, *p* = 0.020], and the global level of functioning [CGAS: *r*_(63)_ = −0.266, *p* = 0.035]. The later findings are illustrated in Fig. 5. Findings suggest that patients with greater depression severity, greater difficulties in emotion regulation and lower functioning showed the greatest effects of tVNS.

## Discussion

The present study is the first to investigate the effects of acute tVNS on emotion recognition in adolescents with MDD. tVNS had no robust effects on self-reports of mood or affect and no effects on the recognition of static or gradually increasing facial expressions of emotions. The recognition of briefly presented stimuli in the Go/NoGo-task increased under tVNS in controls but decreased in patients with MDD. Additional analyses indicated that beyond group effects, there was a linear association between changes in emotion recognition under tVNS and different continuous measures of depression severity and associated symptoms (difficulties in emotion regulation, general functioning). Sensitivity analyses indicated that tVNS specifically led to a decrease in the recognition of *sad* emotions in patients with MDD. In controls, tVNS increased the recognition of *happy* and *sad* emotions. Whereas tVNS generally improves emotion recognition in controls, it seems to specifically decrease the recognition of emotions of *negative* valence in adolescent MDD.

Findings in non-depressed controls are in line with previous research in adults, suggesting that tVNS enhances the general ability to recognize emotions (Sellaro et al., 2018). However, unlike previous studies presenting static stimuli with no response-time limitation, we found tVNS to only affect early visual processing and subsequent behavioral responses to briefly presented stimuli of emotional valence. Even when controlling for item difficulty in line with previous tVNS studies (Colzato et al., 2017; Sellaro et al., 2018), we were not able to replicate the reported effects of tVNS on emotion recognition in healthy adults. Findings in adolescent patients with MDD suggest an increase of valence-specific errors, such that tVNS resulted in an impairment to recognize stimuli of negative valence. The strength of this effect varied as a linear function of depression severity (interview and self-reports), self-reported difficulties in emotion regulation, and clinician-rated level of functioning. Findings suggest that patients with greater symptom severity may show enhanced benefit from tVNS stimulation. These results highlight a potential therapeutic mechanism of tVNS by reducing the attentional bias toward stimuli of negative valence in MDD (Gotlib & Joormann, 2010). A previous neuroimaging study in healthy adults using carotid stimulation to evoke enhanced parasympathetic activity showed similar effects, such that amygdala activity was exclusively affected by carotid stimulation during the appraisal of fearful (not neutral) faces (Makovac et al., 2015). Somewhat puzzling is the fact that this effect is only seen in patients with MDD. Non-depressed controls showed a general improvement on the Go/NoGo task. This might be due to the specific nature of our control group, as adolescents with TTH might have a general deficit in emotion recognition that improves under tVNS. On the other hand, tVNS may have resulted in a general enhancement of emotion recognition as suggested by others (Colzato et al., 2017; Sellaro et al., 2018) when no prior deficit is present.

It has been shown that antidepressant effects of SSRI administration are attributable to an augmentation of serotonin that enhances pleasant and suppresses unpleasant cortical processing of emotionally valent stimuli (Kemp, Gray, Silberstein, Armstrong, & Nathan, 2004). Although the neurobiological mechanisms of tVNS action are largely unknown, animal studies have shown that VNS increases the firing rate of norepinephrine neurons in the locus coeruleus and serotonin neurons in the dorsal raphe nucleus (Manta, El Mansari, & Blier, 2012). Thus, it is plausible that tVNS exerts its antidepressant action through similar mechanisms of serotonergic action, which remains subject to further investigation. However, here we presented preliminary findings on acute tVNS. Future studies need to address how these could accumulate to meaningful clinical effects following long-term treatment. Neuroimaging studies in adults with MDD receiving 4 weeks of tVNS treatment were able to show that resting state functional connectivity between the right amygdala and left dorsolateral prefrontal cortex was increased in the tVNS compared to the sham condition and that this increase was associated with a reduction in depressive symptoms (Liu et al., 2016). Potentially, the short-term effects that we observed translate to changes in neuroplasticity and functional reorganization following longer time periods of tVNS. Respective studies in adolescents are warranted, addressing the clinical utility of tVNS in child and adolescent psychiatric care.

Multi-modal assessments of autonomic nervous system function showed that acute tVNS was associated with an increase in HRV and a decrease of HR only in controls. The absence of effects in patients with MDD suggests that ANS regulation is not susceptible to acute-tVNS, but may require prolonged therapeutic intervention to show meaningful effects (Koenig et al., 2018). In line with the findings of prior meta-analyses (Kemp et al., 2010; Koenig, Kemp, Beauchaine, Thayer, & Kaess, 2016), patients with MDD from the present sample showed greater HR and lower HRV compared to controls. Findings in non-depressed controls contradict previous studies on the physiological effects of acute tVNS that showed no effects of left-sided but right-sided stimulation on HR/HRV in healthy adults (De Couck et al., 2017). Differences between studies are potentially attributable to different stimulation parameters (i.e. Hz, duration; De Couck et al., 2017) that require systematic investigation in future trials. The present findings in non-depressed controls suggest that stimulation of the left vagal nerve tract has small efferent effects on heart function. Effects are characterized by an increase in parasympathetic vagal activity, whereas we found no evidence for changes in sympathetic activity.

The study has some limitations that need to be considered. First, due to legal restrictions, we were not able to recruit a control group of adolescents free of any medical condition. Although the control group consisted of adolescents without depressive symptoms, we cannot rule out the possibility that TTH itself is associated with impaired emotion recognition. TTH has been shown to be associated with emotional distress (Perozzo et al., 2005) and is frequently comorbid with depressive symptoms and anxiety (Song et al., 2016). However, we excluded controls with clinically relevant symptoms of depression, and significant group differences on all clinical variables illustrate that we were able to recruit a non-depressed control group to contrast the clinical phenotype of interest. Comparisons against a completely healthy control group – free of any medical condition – might have shown even stronger effects. Thus, the nature of our control group may even enhance confidence regarding the reported effects. Potentially, the nature of the control group and the age range studied are two reasons why we were not able to replicate previous findings of tVNS on emotion recognition reported in healthy adults (Colzato et al., 2017; Sellaro et al., 2018). Finally, within this limited sample of adolescents, we were not able to address potential mediating effects of sex, medication status, or other confounds.

## Conclusion

To conclude, the present results lend initial support for a potential antidepressant effect of acute tVNS in adolescents with MDD. Future clinical studies are needed, investigating the clinical effects of long-term stimulation on clinical outcomes of depression severity. Further, neuroimaging studies are warranted, addressing potential alterations in cortical processing of emotional stimuli under acute tVNS.

## Footnotes

† The notes appear after the main text.

## Notes

1 Oxygenation of the prefrontal cortex was continuously recorded using an eight-channel functional fNIRS system (OctaMon, Artinis Medical Technologies, BV, The Netherlands). fNIRS data are not reported in the present manuscript.

2 Additional analyses based on reviewer recommendations.

## Supplementary material

For supplementary material accompanying this paper visit https://doi.org/10.1017/S0033291719003490. click here to view supplementary material

## References

[ref1] Aaronson S. T., Sears P., Ruvuna F., Bunker M., Conway C. R., Dougherty D. D., … Zajecka J. M. (2017). A 5-year observational study of patients with treatment-resistant depression treated with vagus nerve stimulation or treatment as usual: Comparison of response, remission, and suicidality. American Journal of Psychiatry, 174, 640–648. 10.1176/appi.ajp.2017.16010034 28359201

[ref2] Beck A., Steer R., & Brown K. (1996). Beck depression inventory – revised. Texas: Harcourt Brace.

[ref3] Birmaher B., & Brent D. (2007). Practice parameter for the assessment and treatment of children and adolescents with depressive disorders. Journal of the American Academy of Child & Adolescent Psychiatry, 46, 1503–1526. 18049300 10.1097/chi.0b013e318145ae1c

[ref4] Breslau J., Gilman S. E., Stein B. D., Ruder T., Gmelin T., & Miller E. (2017). Sex differences in recent first-onset depression in an epidemiological sample of adolescents. Translational Psychiatry, 7, e1139. 28556831 10.1038/tp.2017.105 PMC5534940

[ref5] Burger A. M., Verkuil B., Van Diest I., Van der Does W., Thayer J. F., & Brosschot J. F. (2016). The effects of transcutaneous vagus nerve stimulation on conditioned fear extinction in humans. Neurobiology of Learning and Memory, 132, 49–56. 27222436 10.1016/j.nlm.2016.05.007

[ref6] Colzato L. S., Sellaro R., & Beste C. (2017). Darwin revisited: The vagus nerve is a causal element in controlling recognition of other's emotions. Cortex; A Journal Devoted to the Study of the Nervous System and Behavior, 92, 95–102. 28460255 10.1016/j.cortex.2017.03.017

[ref7] Dalili M. N., Penton-Voak I. S., Harmer C. J., & Munafò M. R. (2015). Meta-analysis of emotion recognition deficits in major depressive disorder. Psychological Medicine, 45, 1135–1144. 25395075 10.1017/S0033291714002591 PMC4712476

[ref8] De Couck M., Cserjesi R., Caers R., Zijlstra W. P., Widjaja D., Wolf N., … Gidron Y. (2017). Effects of short and prolonged transcutaneous vagus nerve stimulation on heart rate variability in healthy subjects. Autonomic Neuroscience: Basic & Clinical, 203, 88–96. 28017263 10.1016/j.autneu.2016.11.003

[ref9] Ebner N. C., Riediger M., & Lindenberger U. (2010). FACES – a database of facial expressions in young, middle-aged, and older women and men: Development and validation. Behavior Research Methods, 42, 351–362. 20160315 10.3758/BRM.42.1.351

[ref10] Fang J., Rong P., Hong Y., Fan Y., Liu J., Wang H., … Kong J. (2016). Transcutaneous vagus nerve stimulation modulates default mode network in major depressive disorder. Biological Psychiatry, 79, 266–273. 25963932 10.1016/j.biopsych.2015.03.025 PMC4838995

[ref11] Frangos E., Ellrich J., & Komisaruk B. R. (2015). Non-invasive access to the vagus nerve central projections via electrical stimulation of the external ear: fMRI evidence in humans. Brain Stimulation, 8, 624–636. 25573069 10.1016/j.brs.2014.11.018 PMC4458242

[ref12] Godlewska B. R., Browning M., Norbury R., Cowen P. J., & Harmer C. J. (2016). Early changes in emotional processing as a marker of clinical response to SSRI treatment in depression. Translational Psychiatry, 6, e957. 27874847 10.1038/tp.2016.130 PMC5314109

[ref13] Gotlib I. H., & Joormann J. (2010). Cognition and depression: Current status and future directions. Annual Review of Clinical Psychology, 6, 285–312. 10.1146/annurev.clinpsy.121208.131305 PMC2845726 20192795

[ref14] Gratz K. L., & Roemer L. (2004). Multidimensional assessment of emotion regulation and dysregulation: Development, factor structure, and initial validation of the difficulties in emotion regulation scale. Journal of Psychopathology and Behavioral Assessment, 26, 41–54.

[ref15] Harmer C. J., Bhagwagar Z., Perrett D. I., Völlm B. A., Cowen P. J., & Goodwin G. M. (2003). Acute SSRI administration affects the processing of social cues in healthy volunteers. Neuropsychopharmacology, 28, 148–152. 12496951 10.1038/sj.npp.1300004

[ref16] Jennings J. R., Kamarck T., Stewart C., Eddy M., & Johnson P. (1992). Alternate cardiovascular baseline assessment techniques: Vanilla or resting baseline. Psychophysiology, 29, 742–750. 1461961 10.1111/j.1469-8986.1992.tb02052.x

[ref17] Kemp A. H., Gray M. A., Silberstein R. B., Armstrong S. M., & Nathan P. J. (2004). Augmentation of serotonin enhances pleasant and suppresses unpleasant cortical electrophysiological responses to visual emotional stimuli in humans. NeuroImage, 22, 1084–1096. 15219580 10.1016/j.neuroimage.2004.03.022

[ref18] Kemp A. H., Quintana D. S., Gray M. A., Felmingham K. L., Brown K., & Gatt J. M. (2010). Impact of depression and antidepressant treatment on heart rate variability: A review and meta-analysis. Biological Psychiatry, 67, 1067–1074. 20138254 10.1016/j.biopsych.2009.12.012

[ref19] Koenig J., Kemp A. H., Beauchaine T. P., Thayer J. F., & Kaess M. (2016). Depression and resting state heart rate variability in children and adolescents – a systematic review and meta-analysis. Clinical Psychology Review, 46, 136–150. 27185312 10.1016/j.cpr.2016.04.013

[ref20] Koenig J., Westlund Schreiner M., Klimes-Dougan B., Ubani B., Mueller B. A., Lim K.O., … Cullen K. R. (2018). Increases in orbitofrontal cortex thickness following antidepressant treatment are associated with changes in resting state autonomic function in adolescents with major depression – preliminary findings from a pilot study. Psychiatry Research. Neuroimaging, 281, 35–42. 30216863 10.1016/j.pscychresns.2018.08.013 PMC6204080

[ref21] Laux L., Glanzmann P., Schaffner P., & Spielberger C. (1981). Das state-trait-angstinventar. Theoretische grundlagen und handanweisung. Weinheim: Beltz.

[ref22] Liu J., Fang J., Wang Z., Rong P., Hong Y., Fan Y., … Kong J. (2016). Transcutaneous vagus nerve stimulation modulates amygdala functional connectivity in patients with depression. Journal of Affective Disorders, 205, 319–326. 27559632 10.1016/j.jad.2016.08.003

[ref23] Makovac E., Garfinkel S. N., Bassi A., Basile B., Macaluso E., Cercignani M., … Critchley H. (2015). Effect of parasympathetic stimulation on brain activity during appraisal of fearful expressions. Neuropsychopharmacology, 40, 1649–1658. 25578794 10.1038/npp.2015.10 PMC4915246

[ref24] Manta S., El Mansari M., & Blier P. (2012). Novel attempts to optimize vagus nerve stimulation parameters on serotonin neuronal firing activity in the rat brain. Brain Stimulation, 5, 422–429. 22037140 10.1016/j.brs.2011.04.005

[ref25] Osman A., Kopper B. A., Barrios F., Gutierrez P. M., & Bagge C. L. (2004). Reliability and validity of the Beck Depression Inventory – II with adolescent psychiatric inpatients. Psychological Assessment, 16, 120–132. 15222808 10.1037/1040-3590.16.2.120

[ref26] Peirce J. W. (2007). Psychopy – psychophysics software in Python. Journal of Neuroscience Methods, 162, 8–13. 17254636 10.1016/j.jneumeth.2006.11.017 PMC2018741

[ref27] Perozzo P., Savi L., Castelli L., Valfrè W., Lo Giudice R., Gentile S., … Pinessi L. (2005). Anger and emotional distress in patients with migraine and tension-type headache. The Journal of Headache and Pain, 6, 392–399. 16362712 10.1007/s10194-005-0240-8 PMC3452065

[ref28] Polanczyk G. V., Salum G. A., Sugaya L. S., Caye A., & Rohde L. A. (2015). Annual research review: A meta-analysis of the worldwide prevalence of mental disorders in children and adolescents. Journal of Child Psychology and Psychiatry, and Allied Disciplines, 56, 345–365. 10.1111/jcpp.12381 25649325

[ref29] Poznanski E., Freman L., & Mokros H. (1985). Children's depression rating scale-revised. Psychopharmacology Bulletin, 21, 979–989.

[ref30] Sellaro R., de Gelder B., Finisguerra A., & Colzato L. S. (2018). Transcutaneous vagus nerve stimulation (tVNS) enhances recognition of emotions in faces but not bodies. Cortex, 99, 213–223. 29275193 10.1016/j.cortex.2017.11.007

[ref31] Shaffer D., Gould M. S., Brasic J., Ambrosini P., Fisher P., Bird H., & Aluwahlia S.(1983). A children's global assessment scale (CGAS). Archives of General Psychiatry, 40, 1228–1231. 6639293 10.1001/archpsyc.1983.01790100074010

[ref32] Sheehan D. V., Sheehan K. H., Shytle R. D., Janavs J., Bannon Y., Rogers J. E., … Wilkinson B. (2010). Reliability and validity of the Mini International Neuropsychiatric Interview for Children and Adolescents (MINI-KID). The Journal of Clinical Psychiatry, 71, 313–326. 20331933 10.4088/JCP.09m05305whi

[ref33] Song T.-J., Cho S.-J., Kim W.-J., Yang K. I., Yun C.-H., & Chu M. K. (2016). Anxiety and depression in tension-type headache: A population-based study. PLoS ONE, 11, 1–12. 10.1371/journal.pone.0165316 PMC5082613 27783660

[ref34] Trinkl M., Greimel E., Bartling J., Grünewald B., Schulte-Körne G., & Grossheinrich N. (2015). Right-lateralization of N2-amplitudes in depressive adolescents: An emotional go/no-go study. Journal of Child Psychology and Psychiatry, 56, 76–86. 24963551 10.1111/jcpp.12282

[ref35] Vos T., Allen C., Arora M., Barber R. M., Bhutta Z. A., Brown A., … Murray C. J. L. (2016). Global, regional, and national incidence, prevalence, and years lived with disability for 310 diseases and injuries, 1990–2015: A systematic analysis for the Global Burden of Disease Study 2015. The Lancet, 388, 1545–1602. 10.1016/S0140-6736(16)31678-6 PMC5055577 27733282

[ref36] Watson D., Clark L. A., & Tellegen A. (1988). Development and validation of brief measures of positive and negative affect: The PANAS scales. Journal of Personality and Social Psychology, 54, 1063–1070. 3397865 10.1037//0022-3514.54.6.1063

