# Supplementary figures and images for: Effects of acute transcutaneous vagus nerve stimulation on emotion recognition in adolescent depression
Source: Psychol Med. 2019 Dec 10;51(3):511–20. doi: 10.1017/S0033291719003490 (PMC7958483; doi:10.1017/S0033291719003490)

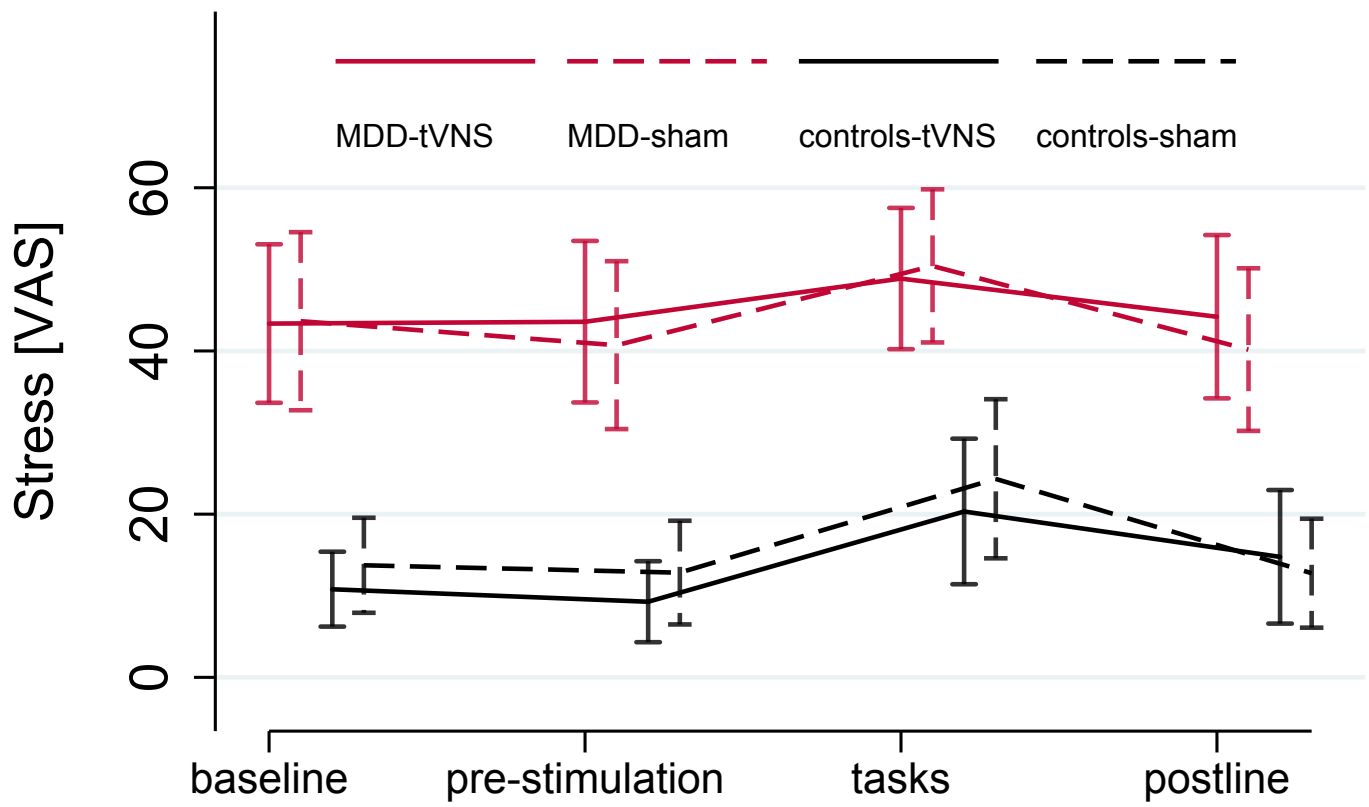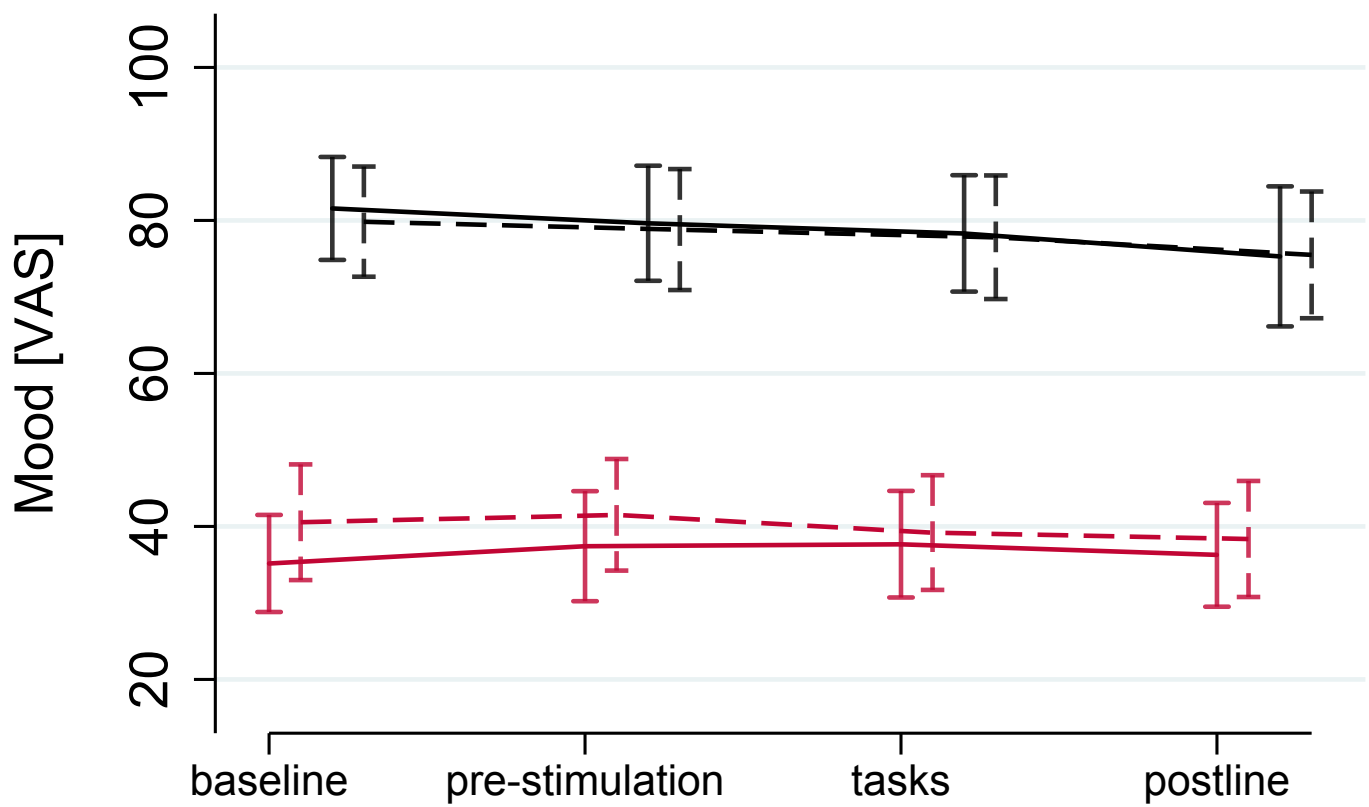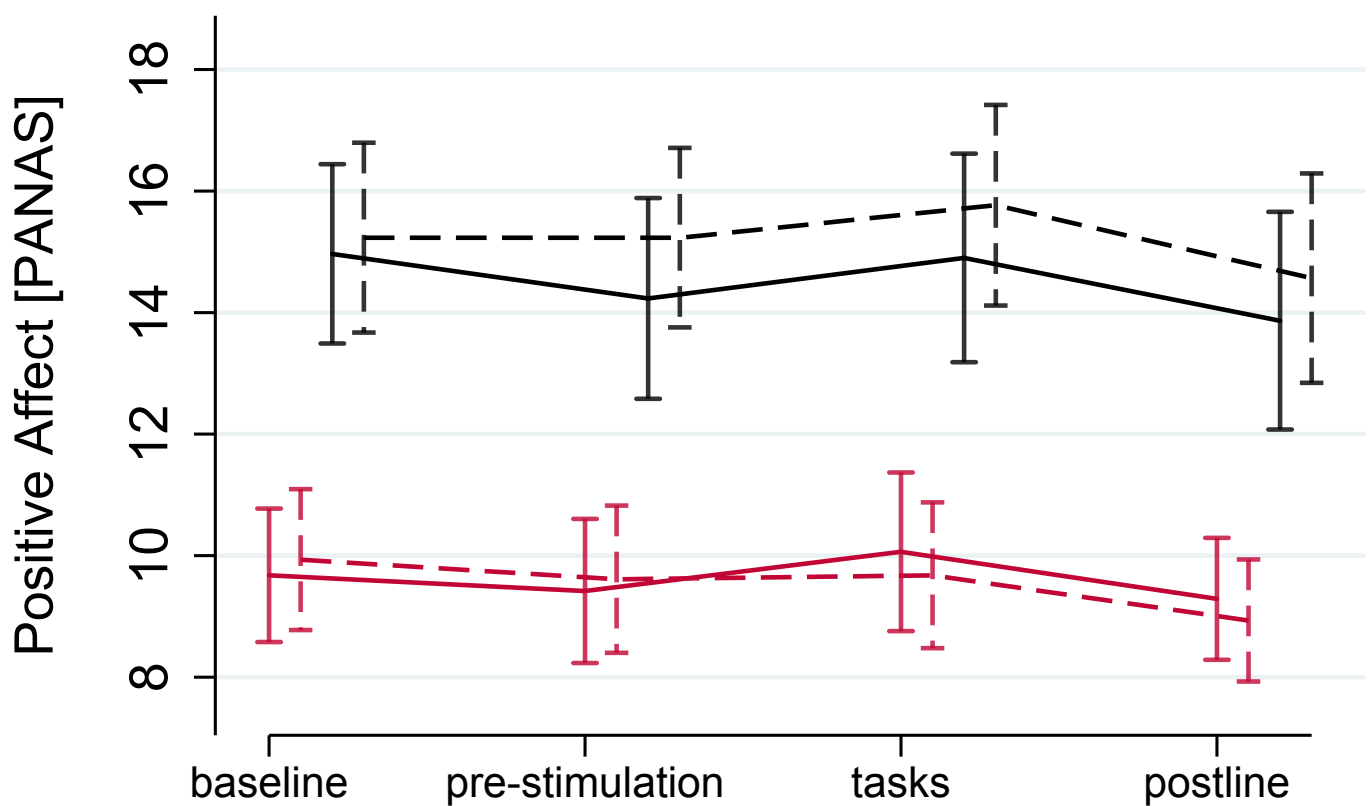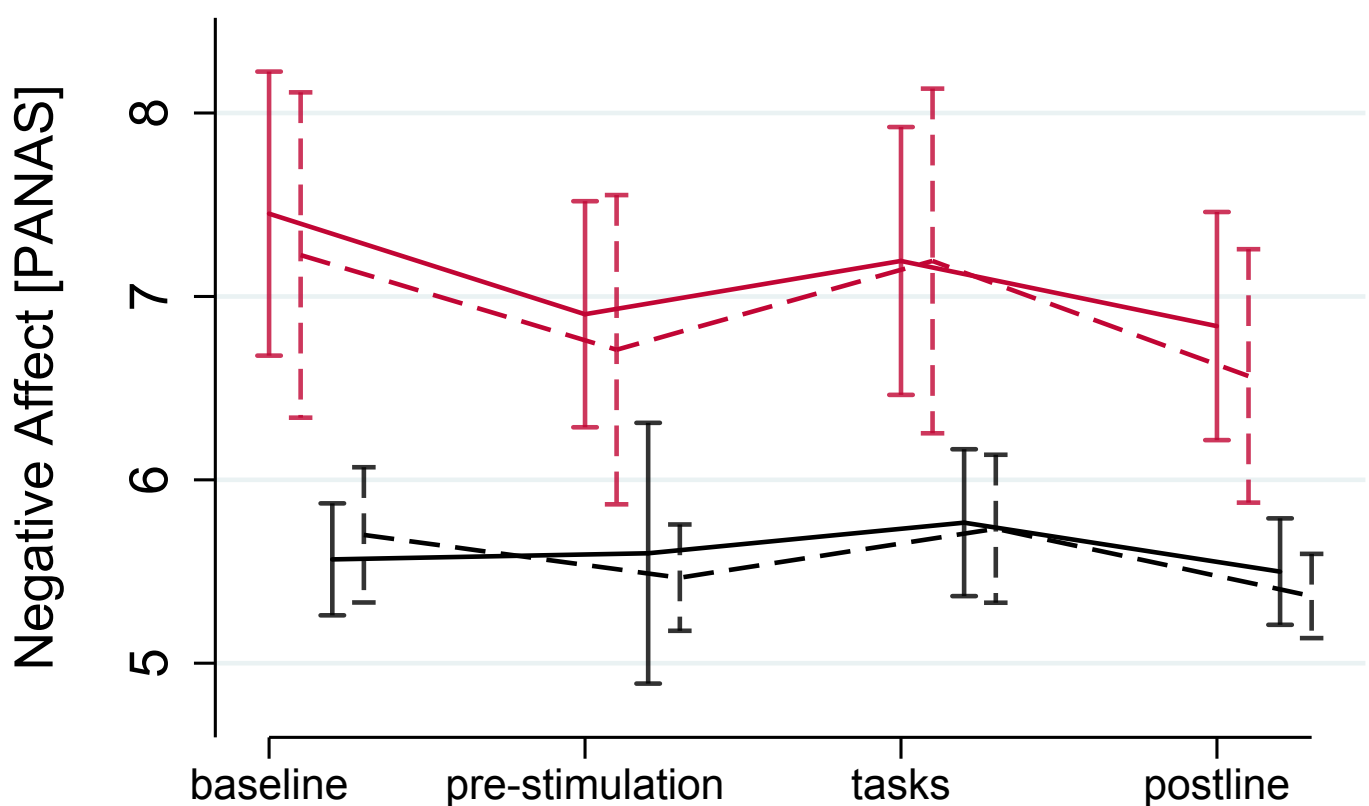

Supplement: Supplementary file 1 [file S0033291719003490sup.zip › S0033291719003490sup001.pdf]

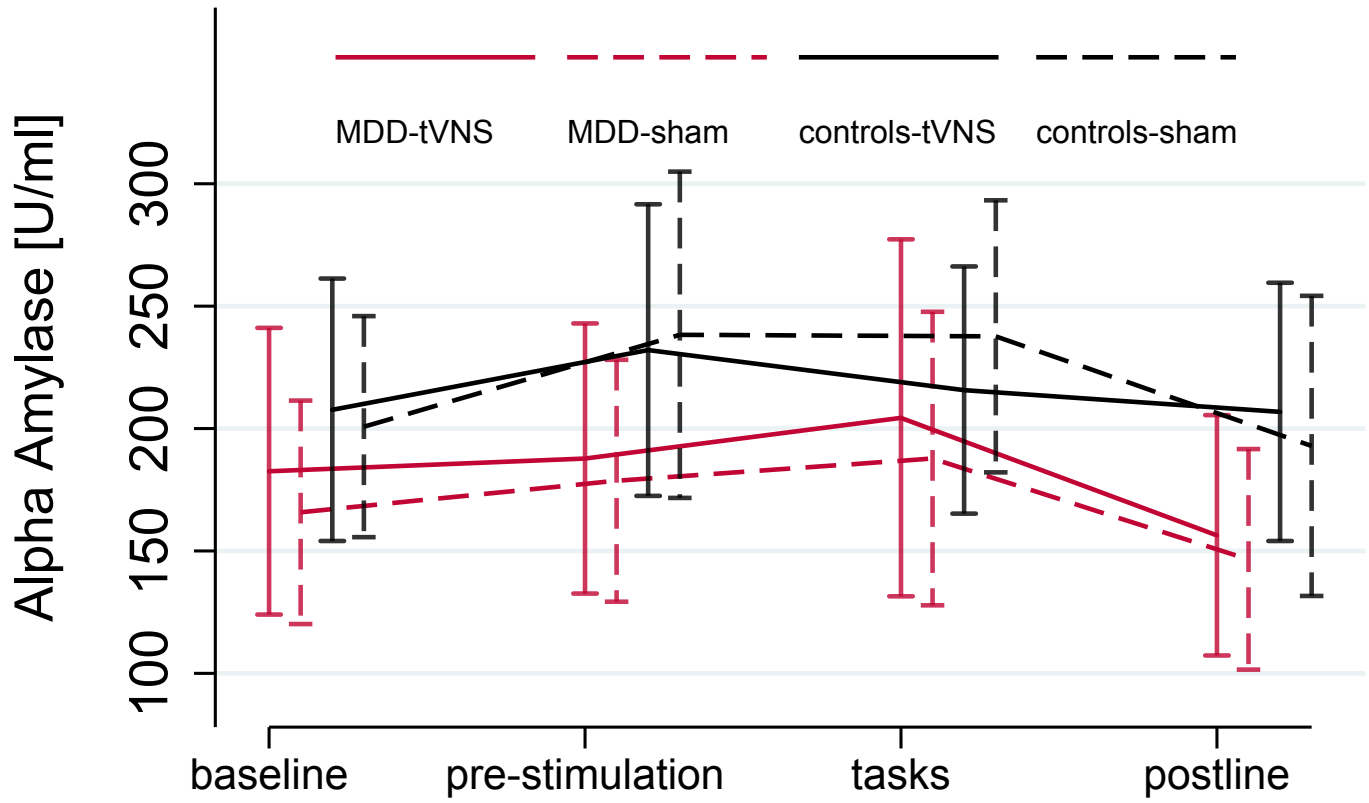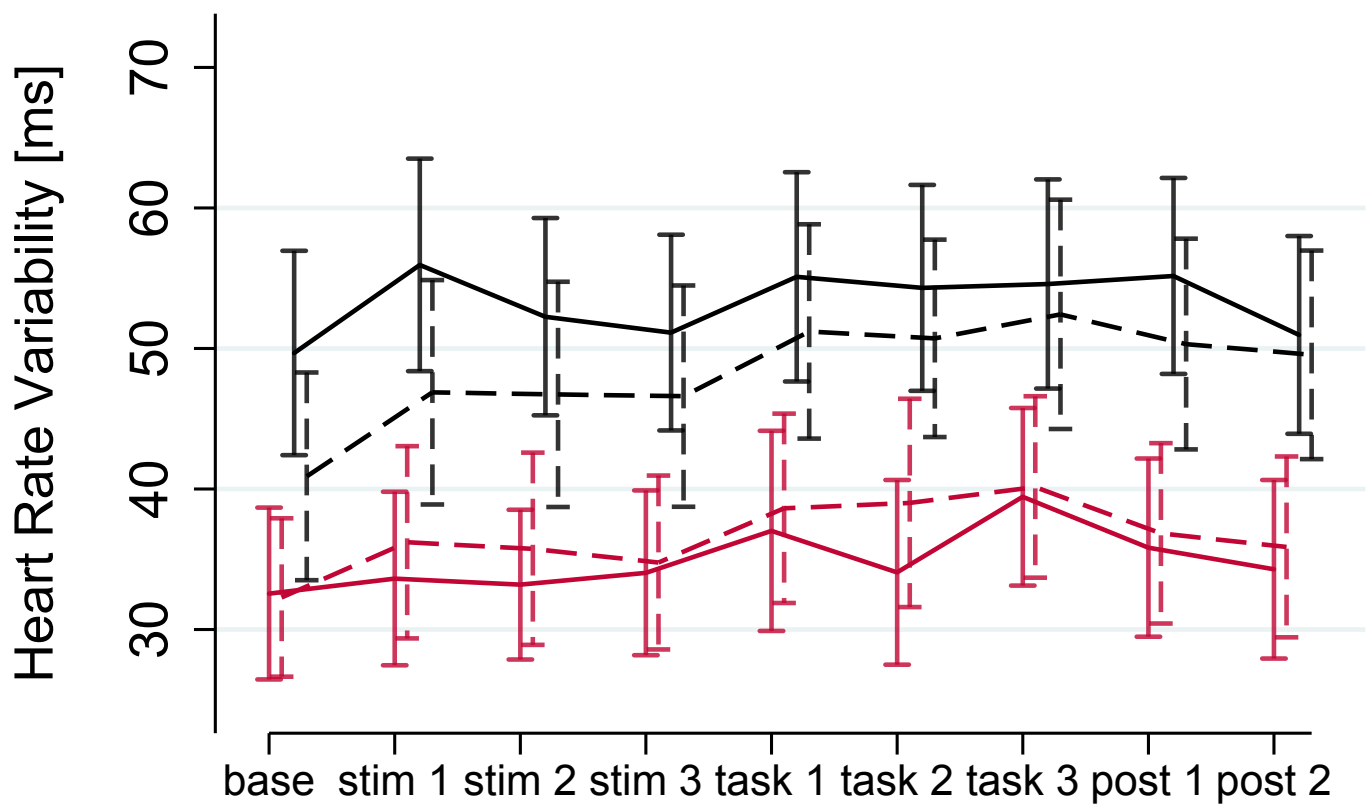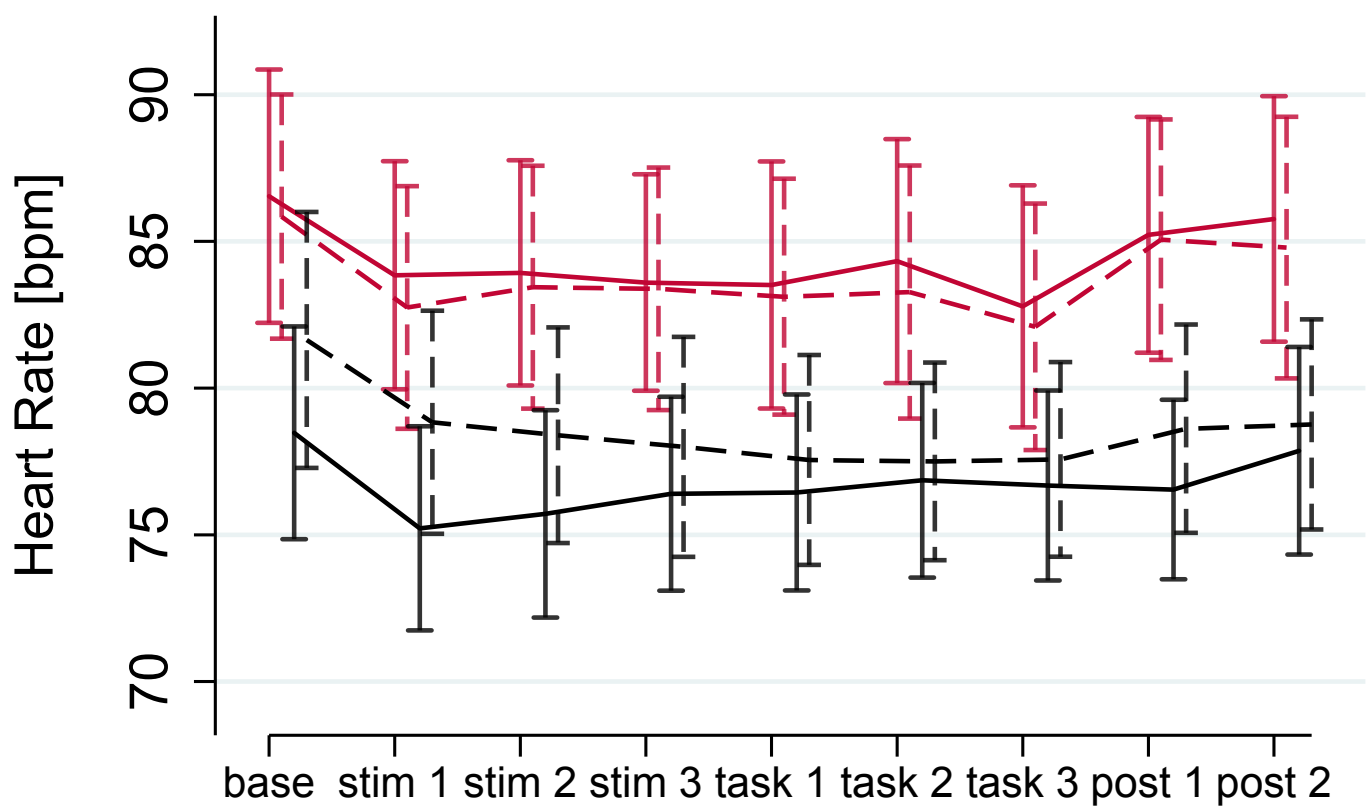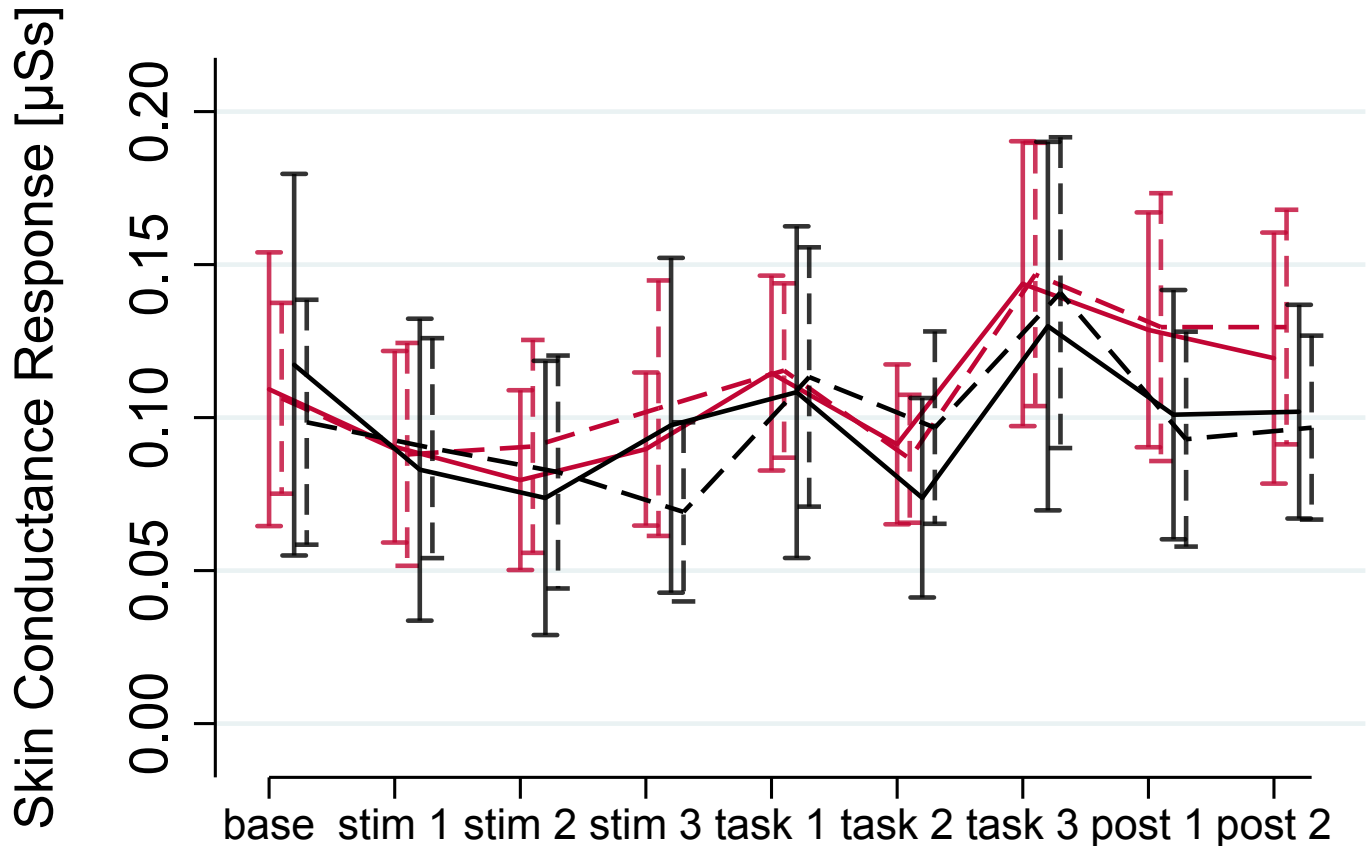

Supplement: Supplementary file 1 [file S0033291719003490sup.zip › S0033291719003490sup002.pdf]
